# "Design of a Suspension Lever Mechanism in Biomedical Robotic System"

**DOI:** 10.3389/frobt.2022.906691

**Published:** 2022-07-22

**Authors:** A. Voloshkin, A. Tereshchenko, G. Carbone, L. Rybak, A. Nozdracheva

**Affiliations:** ^1^ Belgorod State Technological University Named After V. G. Shukhov, Belgorod, Russia; ^2^ Department of Mechanical Engineering, Energy Engineering and Management, University of Calabria, Rende, Italy

**Keywords:** parallel robots, orthosis position, kinematic analysis, lever system, elastic elements, medical robot

## Abstract

The article discusses the design of a suspended lever mechanism with elastic elements, which is used as a safety device in a robotic system for the rehabilitation of the lower limbs. The article analyzes the existing mechanical structures of devices for rehabilitation, identifies the problems of operation, design, and safety systems and suggests a new design of the device. The process of reverse development of a lever mechanism scheme to ensure safety during rehabilitation of the lower limbs is presented. The design of the lever mechanism consists of movable levers connected by elastic elements. The device allows you to dampen the force during active rehabilitation. The power calculation of the lever mechanism in the rehabilitation system was carried out. The article addresses the issues present in the current mechanical designs with a brief discussion on the system architecture.

## 1 Introduction

The article discusses methods of increasing the effectiveness of mechanotherapy in the rehabilitation of the lower limbs through the use of new technical solutions and principles of intelligent robotics. Currently, there are a number of tasks in practical healthcare, the optimal way to solve them is the use of robotic tools. These tasks relate not only to the treatment and rehabilitation of patients with musculoskeletal disorders, but also to the implementation of their self-service functions, social adaptation, and replenishment of lost motor and communication functions. Continuous passive motion (CPM) is one of the most common therapies at the initial stage of treatment, when patients have low or generally uncontrolled limbs. All this requires the introduction of new technical and technological solutions Vashisht and Puliyel (2012); Truelsen and Bonita (2009); Loeb (1989); Stein et al. (1998).

Currently, there are many devices for rehabilitation of the lower limbs “Gait-trainers” (2022); Bouri et al. (2006, 2009); Wang et al. (2020); LokoHelp (2022); Rios et al. (2018); Almaghout et al. (2020); ErigoPro (2022). Among the well–known models of robotic mechanotherapy systems are such as BioDex, Cybex Humac Norm and Ekso Bionics Ekso (all—United States), LOKOMAT®PRO (Switzerland), Amadeo and ReWalk (Israel), Cyberdyne HAL (Japan) Hidler and Wall (2005); Lam et al. (2006); Mirbagheri et al. (2005). In Russia, the production of exercise machines and exoskeletons ORMED and ExoaThlet has been established. Erigo, Lokomat, Lokohelp, Rehabot, GaitTrainer, Lopes and other robotic devices are used to restore the locomotor function of the entire lower limb. In mechanotherapy, the restoration of the patient’s motor function is carried out due to the moving elements of the rehabilitation system. It is important to ensure safety for human health in the event of an unforeseen situation, both with the help of a software and hardware complex and with the help of additional mechanical devices Yoshihiro and Ka (2014). Safe interaction in the robot-human system can be ensured through the use of collaborative robots (cobots) Cherubini et al. (2016); Galin et al. (2020); Ermishin and Yuschenko (2016); Zanchettin et al. (2016); Hoffman (2019); Krüchten and Schadschneider (2017). The described principles of cobots involve the use of sensors, encoders, technical vision, and the manipulator stops based on a software algorithm. Such a robot-human system can be implemented by a mechanical device, parts of which will dampen the impact of the manipulator in a collision or jamming. The mechanical part of such protection can be made in the form of a suspended lever mechanism with elastic elements that will deform under load exceeding the safe threshold of the patient’s physiological capabilities. For the most part, lever mechanisms with elastic elements are used to compensate for the load in dynamic systems Khalilov et al. (2017); Gerrard (2002); Nasada (2006). The article proposes a design of a safety lever mechanism for a robotic system of rehabilitation of the lower limbs.

This document is organized as follows: Section 2 describes the design of the system for the rehabilitation of the lower limbs, describes the process of creating an individual passive orthosis and its digital counterpart, proposes a scheme of a suspended lever mechanism built in the context of assembling a virtual environment; then Section 3 describes the basic mathematical model of the proposed device, describes numerical calculation. Section 4 presents the results of calculations, provides an analysis of the results and presents the final design of the device being developed.

## 2 Orthosis design and development of a suspension lever mechanism scheme

**FIGURE 1:**
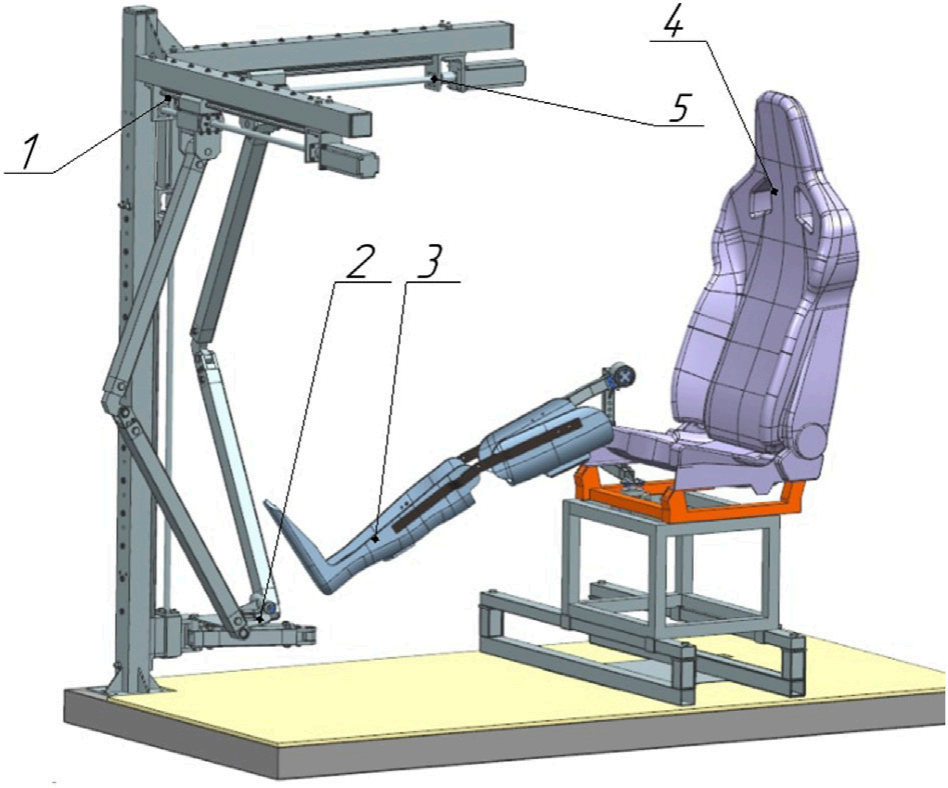
Robotic system for rehabilitation of the lower limbs. 1—active 3-PRRR manipulator, 2—mobile platform, 3—passive orthosis, 4—chair, 5—drive mechanisms.

In previous works Ahmetzhanov et al. (2022); Voloshkin et al. (2020), the design of a robotic system (RS) for the rehabilitation of the patient’s lower limbs was considered. The RS consists of an active 3-PRRR manipulator for moving the patient’s foot fixed on a movable platform, and a serial RRRR manipulator used as a passive orthosis to support the patient’s lower limb fixed on an adjustable chair Figure 1. The robot’s drive mechanisms are made in the form of ball-screw gears.

**FIGURE 2:**
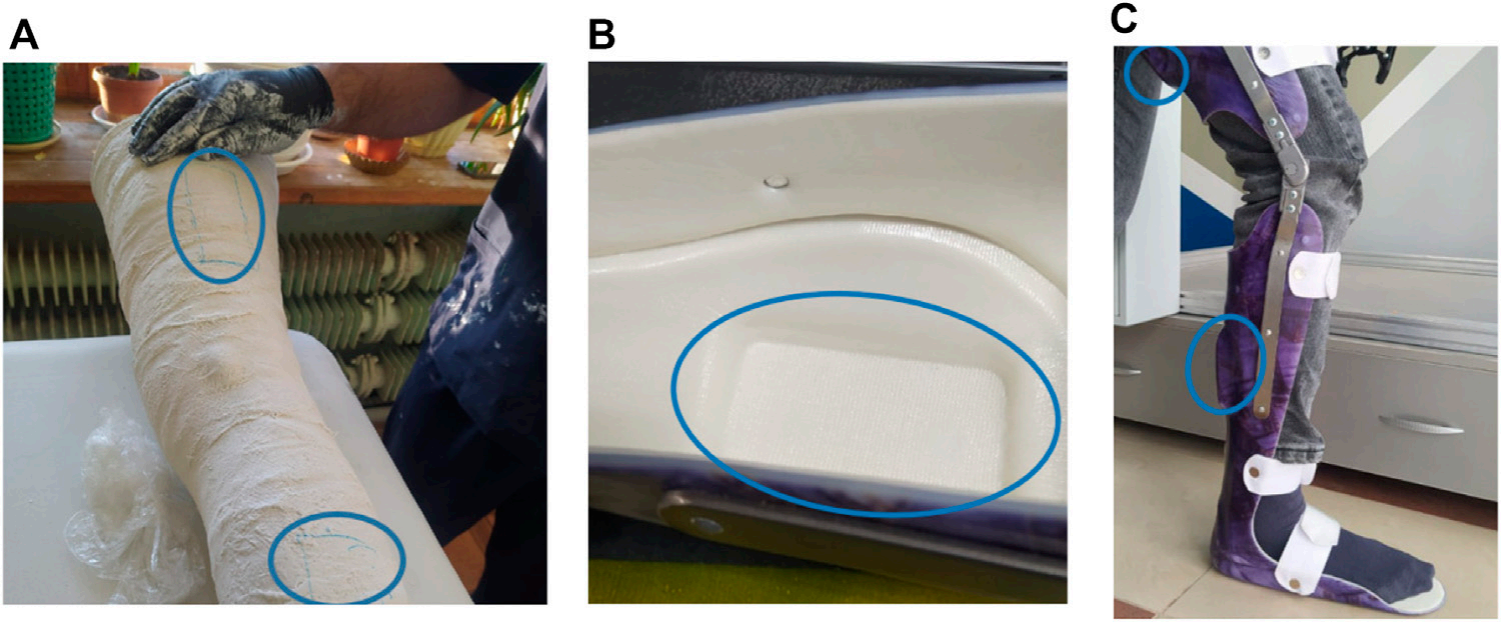
Orthosis production: **(A)**—formation of a plaster cast of the leg of the subject, **(B)**—a groove for fastening, **(C)**—a ready orthosis with marked sites for fastening.

Rehabilitation of the patient in two planes is a difficult task and to minimize errors at the stage of the prototype of the RS, an individual orthosis made in accordance with the anthropometric data of the patient will be used. This will eliminate errors in the construction of the joints of the passive orthosis and reduce the risks during further testing. To develop a more accurate digital layout, an orthosis was made based on a plaster cast of the subject’s leg with marks of the intended attachment in the form of a rectangle (Figure 2A). At the orthosis forming stage, inserts were added to attach the suspension lever mechanism with elastic elements. When the vacuum molding was performed, rectangular grooves were formed on the surface of the orthosis for the intended attachment (Figures 2B,C).

**FIGURE 3:**
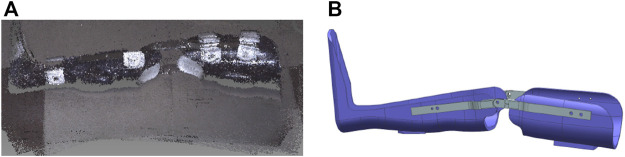
Developing a digital orthosis double: **(A)**—a 3D model obtained using a scanner, **(B)**—a processed 3D orthosis model.

After fitting the manufactured orthosis to the test, a digital double was developed using digital copying methods (Figure 3A). An STL file was obtained using the CREALITY 3D scanner. It was uploaded to the CAD system workspace (Figure 3B) for further solid-state RS modeling.

The drive mechanisms of the RS have high rigidity and complicate feedback by reading information from the motors. At the same time, the RS provides the required effort for rehabilitation, which can injure the patient when the limb falls into a position not provided for by physiology. Therefore, the applied effort during rehabilitation is supposed to be controlled by data from the controller, encoders, sensors of muscle activity and the proposed suspension lever mechanism with elastic elements. The mechanism is used to connect a passive orthosis and an active manipulator and provides cushioning of a dangerous load for the patient during rehabilitation.

**FIGURE 4:**
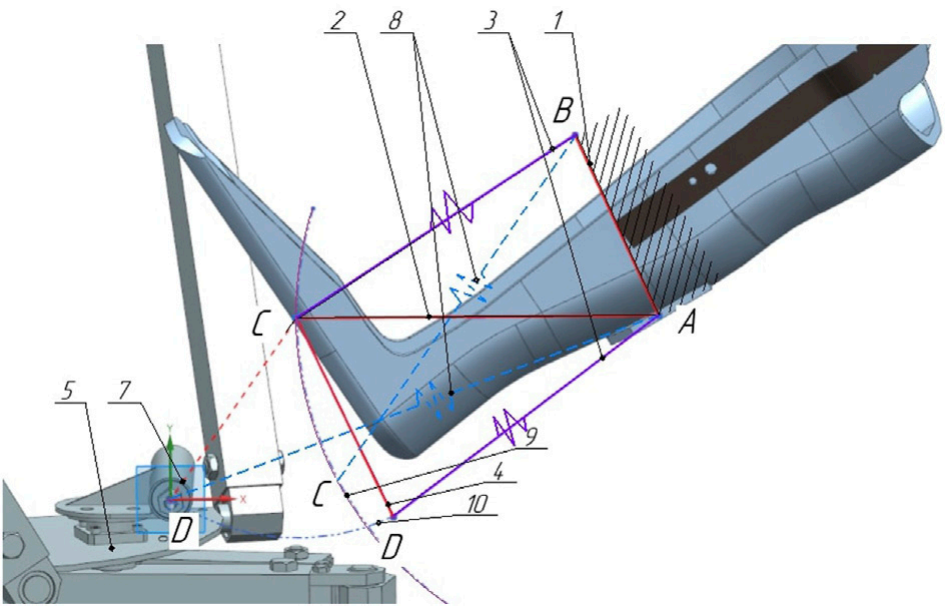
Layout of the links of the suspension lever mechanism. 1—base, 2 - horisontal lever, 3—elastic elements, 4—movable lever, 5—movable platform.

Figure 4 shows a diagram of a suspended lever mechanism with a display of the compressed and initial state. The mount is the base on which the horizontal lever and elastic elements are attached, while one of them is connected to the mount and the other to the movable lever. The horizontal lever and the movable lever are connected pivotally at point C. The lever is connected to the movable platform of the robot and the elastic element connected to the horizontal lever at point A. A suspended lever mechanism with elastic elements is depicted in a folded state, this allows you to determine the extreme deformable position of the elastic elements, provided that the mechanism does not collide with the orthosis. Since the elastic elements have the same compression parameters, we will depict in the diagram (Figure 4) the lever four in the initial position 7, while the elastic elements will take the position 8. The trajectories of the rotation of the levers are indicated by positions 9 and 10. We will get the required angles and lengths of the levers based on the size of the orthosis.

**FIGURE 5:**
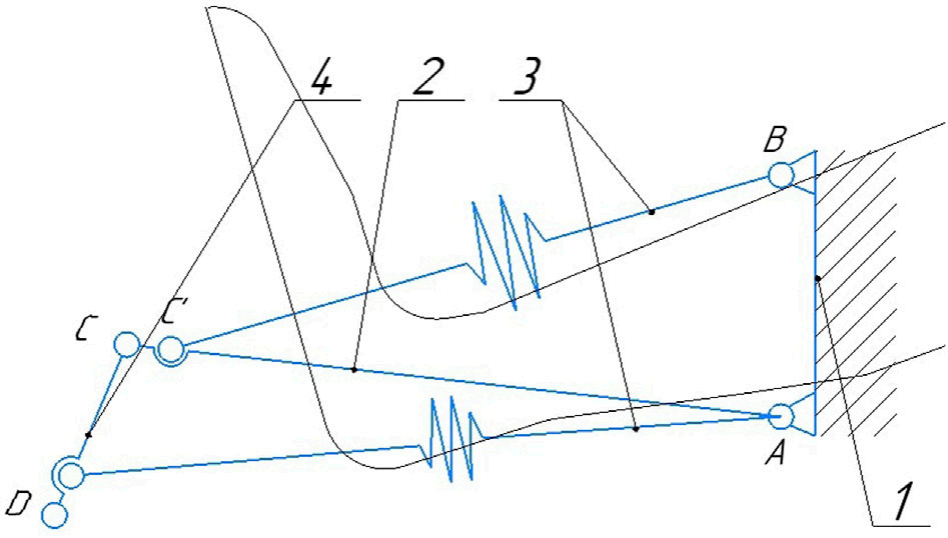
Diagram of a suspended lever mechanism with elastic elements 1—fastening of the orthosys, 2—horisontal lever, 3—elastic elements, 4—movable lever.

Joints A and B are rigidly fixed on the ankle part of the orthosis, which we consider conditionally fixed (Figure 5). The point of application of force from the side of the active manipulator is located in the center of the joint D. As a result of the force, reversible deformation (compression) of the elastic elements will occur, due to the rotation of the levers, while the fastening of the orthosis will remain stationary. Fastening of elastic elements and levers, occurs by means of joints *C*′ and *D*′, respectively.

The suspension lever mechanism with elastic elements is arranged in such a way that the elastic elements in the initial (undeformed) position exert maximum compression resistance, since the angle of application of external force as the elastic element is compressed will increase, as will the effect of force. Such a structure of the mechanism allows us to assert that an elastic element with constant rigidity is predictably deformed at a given load. In this case, the movement to the extreme position of the deformation of the elastic element will occur without increasing the load on the patient’s limb.

## 3 Calculation of a suspended lever mechanism with elastic elements

Based on the constructed structural diagram of a suspended lever mechanism with elastic elements, we will draw up a calculation scheme to determine the dependence of the applied load and the compression force of elastic elements Woo and Freudenstein (1970). Let’s determine the number of degrees of freedom of the mechanism by Chebyshev’s formula,

$$W=3n-2p_{1}=3\cdot 2-2\cdot 2=2$$

**FIGURE 6:**
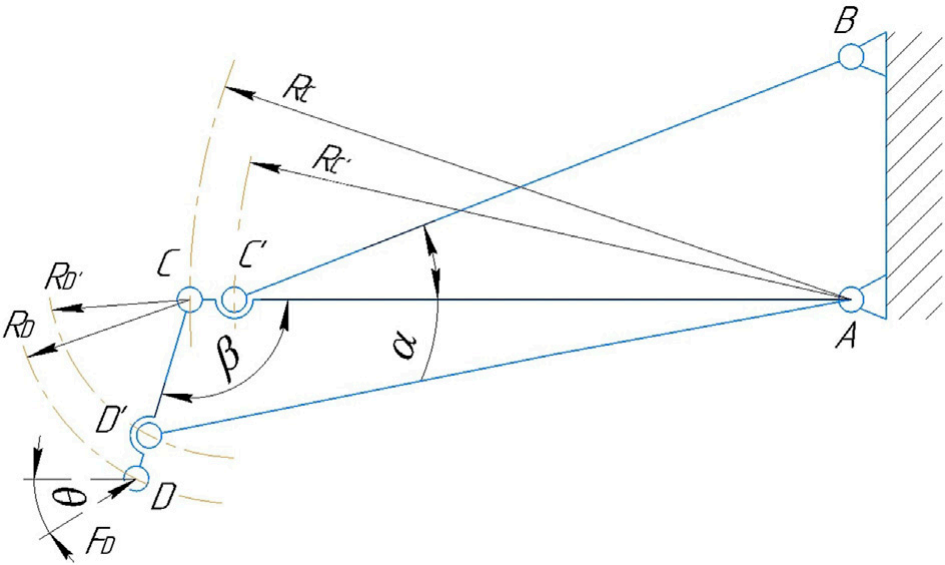
Design scheme of the suspension lever mechanism.

The system has two degrees of mobility and is undetectable. However, if elastic elements are represented in the initial (not compressed) state in the form of rigid rods, then a movable system with two degrees of mobility can be considered as a static truss (Figure 6). In the diagram, *R*_*C*_ is the radius of rotation of the lever *AC*, $R_{C}^{\prime }$ is the radius of rotation of the point *C*′ on the lever *CA*, *RD* is the radius of rotation points *D* relative to the joint *C*, *RD*′ is the radius of rotation of point *D*′ relative to the joint *C*, and *FD* is the external force, *α* is the angle between the horizontal lever *CA* and the elastic element *C*′*B*, *β* is the angle between the levers *AC* and *CD*.

**FIGURE 7:**
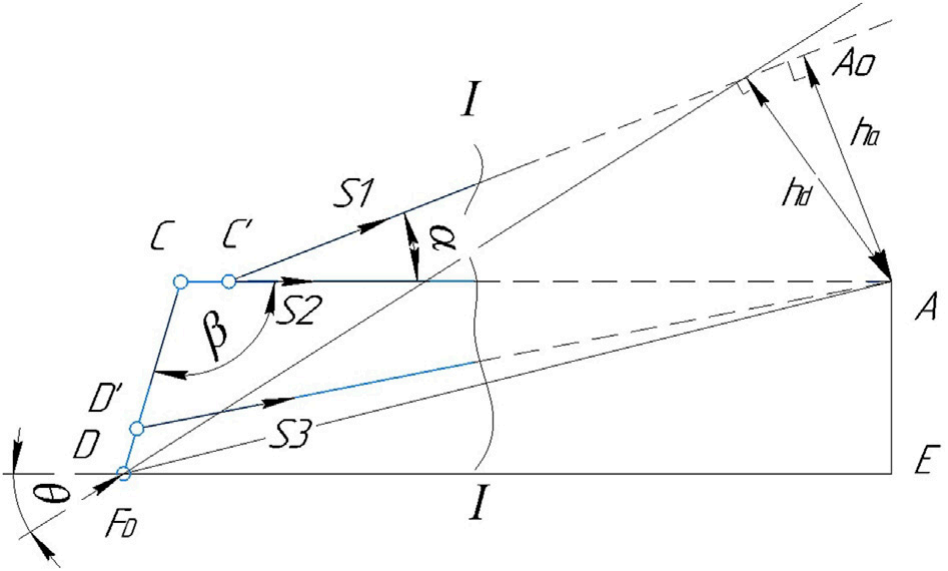
Design diagram of the left part of the suspension lever mechanism.

When determining the effort, all the rods of the truss are considered stretched. It is required to determine the force in the rods *C*′*B* and *D*′*A*. To do this, we carry out the section I-I, dissecting no more than three rods, including *C*′*B* and *D*′*A*, the force in which is determined. We discard the left part of the truss, replacing its action with the remaining left part by the efforts of *S*_1_, *S*_2_ and *S*_3_ applied in the corresponding sections of the rods and directed towards the discarded part (Figure 7).

To determine the force *S*_1_, we make up the equation of the moments of forces acting on the first part of the truss, relative to point A, at which the lines of action of forces *S*_2_ and *S*_3_ intersect. This point is called the Ritter point: *∑M*_*A*_ = 0;

$$-S_{1}h_{a}-F_{D}h_{d}=0,$$ (1)

where *h*_*a*_ is the lever of force *S*_1_, wich can be defined as

$$h_{a}=R_{C}^{\prime }\sin\alpha ,$$ (2)

Let’s define *h*_*d*_—the lever of force *FD*, which can be determined by the following formula:

$$h_{d}=DA\sin\left(\theta -\arcsin\frac{AE}{DA}\right),$$ (3)

where side *AE* is defined as

$$AE=R_{D}\cos\left(90-\beta \right),$$ (4)

and the DA side will be determined by the following formula

$$DA=\sqrt{{\left(-R_{D}\cos\beta +R_{C}\right)}^{2}+{\left(R_{D}\sin\beta \right)}^{2}}$$ (5)

Substituting expressions (Eqs 4, 5) into expression (Eq. 3) we get

$$H_{d}=DA\sin\left(\theta -\arcsin\left(\frac{AE}{DA}\right)\right)$$ (6)

Let’s use the same cross-section I-I to determine the forces of *S*_3_ regardless of the forces of *S*_1_ and *S*_2_. We project all the forces acting on the left side of the truss onto the vertical *Y* axis, since the projection of force *S*_2_ on this axis is zero. If *∑F*_*Y*_ = 0, then

$$F_{D}\cos\theta +s_{3}\sin\left(\beta -90\right)+S_{1}\sin\alpha =0,$$ (7)

Let’s express the forces *S*_1_ and *S*_3_ based on expressions (Eqs 1–7), then

$$\left\{\begin{array}{c} S_{1}=-\frac{F_{D}DA\sin\left(\theta -\arcsin\left(\frac{AE}{DA}\right)\right)}{R_{C}^{\prime }\sin\alpha }\\ S_{3}=-\frac{F_{D}R_{C}^{\prime }\sin\alpha \cos\theta +F_{D}DA\sin\left(\theta -\arcsin\left(\frac{AE}{DA}\right)\right)\sin\alpha }{R_{C}^{\prime }\sin\alpha \cos\beta } \end{array}\right.$$ (8)

Suppose that the patient has passed the initial stage of rehabilitation, and the patient’s limb should not be affected by a force *F*_*D*_ exceeding 70 N Gee and Adejumo (2021). Otherwise, the elastic elements of the suspension lever mechanism dampen the load. The dimensions of the levers will be set in accordance with the dimensions of the digital orthosis model (Figure 4): *R*_*D*_ = 0.153 m, $R_{D}^{\prime }$ = 0.141 m, *R*_*C*_ = 0.247 m, $R_{C}^{\prime }$ = 0.235 m.

**FIGURE 8:**
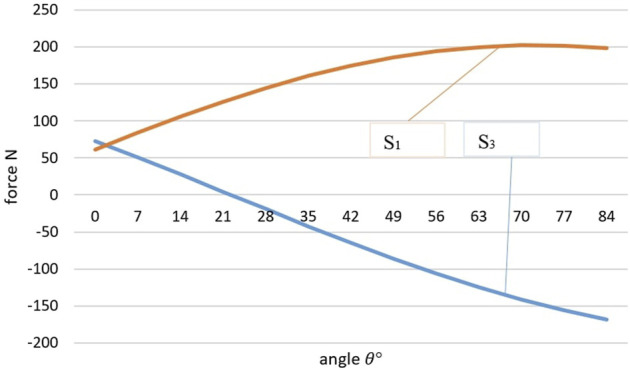
Graph of the dependence of the force acting on elastic elements on the angle of the external force of the active manipulator.

We calculate the loads *S*_1_ and *S*_3_ acting on elastic elements using the Formula (8) at the initial state of the system corresponding to the angle *α* = 33°,*β* = 120°, at different angles *θ*. Figure 8 shows the dependence of the loads *S*_1_ and *S*_3_ on the angle *θ*.

When the angle *θ* changes, the load is redistributed between the elastic elements. The graph shows that the maximum load *S*_1_ = 207.7 N occurs at *θ* = 60°, and the maximum load *S*_3_ = 70 N occurs at *θ* = 0°. At *θ* = 4°, an equal effect of an external force on the elastic elements occurs. It is worth noting that by the effective load on the elastic element, it is possible to determine the direction of the impact of the force of the active manipulator, or set it, if elastic elements are considered as a way to obtain feedback.

**FIGURE 9:**
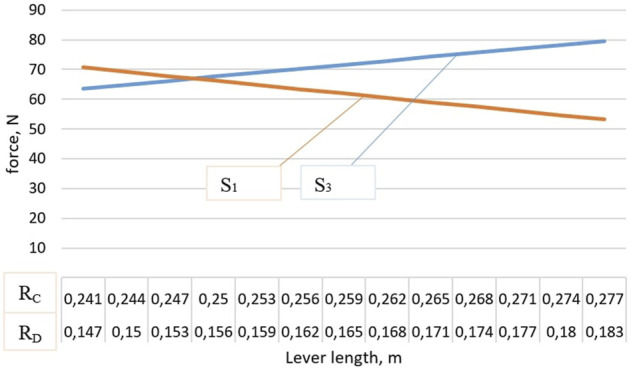
Graph of the dependence of the force acting on elastic elements on the size of the levers.

Figure 9 shows a graph of the dependence of the force acting on elastic elements, depending on the length of the levers, at *θ* = 4°. The increase in each of the levers was made equally, however, increasing the lever AC leads to a significant increase in the force *S*_3_.

**FIGURE 10:**
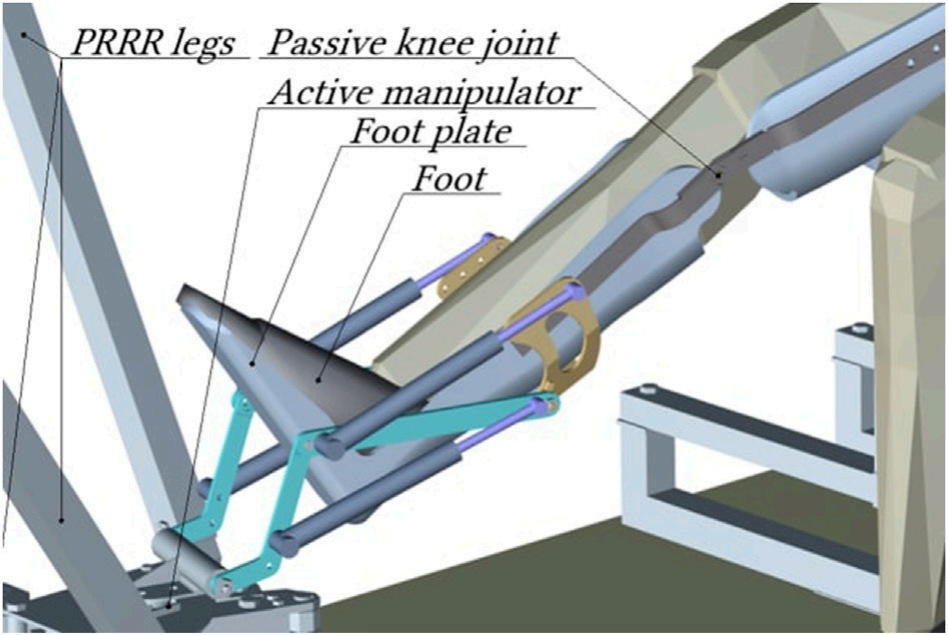
Model of a suspended lever device with elastic elements.

Figure 10 shows a model of a suspended lever mechanism with elastic elements developed using a CAD system. Gas spring is selected as elastic elements, which are compressed at a load of 103.8 and 35 N to stabilize the lever mechanism in space during compression. Elastic elements are installed on each side of the orthosis. The stiffness of the elastic elements is reduced by 2 times.

## 4 Further research

The device of the elastic elements of the suspended lever mechanism can be implemented using hydraulic cylinders, these devices have sufficient smoothness, and their rigidity can be adjusted in real time. During the calculations, a rigid dependence of the angles of rotation of the levers and changes in the length of the elastic elements was revealed. Determination of clear mathematical dependences of changes in the length of elastic elements and the angles of rotation of levers will allow developing a sensitive feedback system for active rehabilitation, which can be done using Lagrange equations of the second kind.

## 5 Conclusion

The kinematic calculation of the suspended lever mechanism with elastic elements justifies the installation of the device in the system for rehabilitation. Based on the calculations, the maximum force must be applied in the initial position of the system, thus, assuming that the stiffness of the elastic element does not change from the initial position to complete deformation, it can be argued that after the system is out of equilibrium, the subsequent deformation requires less load. Such a system does not cause an increase in load during deformation, which means it is possible to set a threshold of the applied force for operation, after passing which there will be no load on the patient’s limb more than calculated. It can be argued that when there is a force on the patient’s limb up to 207.7 N, deformation will not occur only in one elastic element, while the other elastic element is deformed at 70 N. This suggests that it is necessary to install elastic elements of various stiffness, the ratio of which is easily determined from the proportion. Hydraulic cylinders with individual check valves can be one of the options for fine-tuning the stiffness. The suspended lever device will eliminate the disadvantages of rigid robotic systems for rehabilitation, in which feedback is poorly implemented. This will allow the patient to feel safer in the rehabilitation chair, which will significantly increase the effectiveness of exercises. However, the system still does not solve the problem of determining the exact force of the active manipulator on the patient’s limb with variable stiffness of elastic elements. This imposes restrictions on the use of a wide range of standard solutions, which increases the cost of the system.

## Data Availability

The original contributions presented in the study are included in the article/supplementary material, further inquiries can be directed to the corresponding author.
